# Electrochromic, Capacitive, and Electrocatalytic Performance of TEMPO Anchored EDOT-SN(T)S-EDOT Electrode

**DOI:** 10.1021/acsomega.5c02762

**Published:** 2025-07-03

**Authors:** Begüm Nemutlu, Emre Fatih Eker, Ahmet M. Önal, Emine Gul Cansu Ergun, Cihangir Tanyeli

**Affiliations:** † 52984 Middle East Technical University, Department of Chemistry, Ankara 06800, Türkiye; ‡ 37505 Baskent University, Department of Electrical and Electronics Engineering, Ankara 06790, Türkiye

## Abstract

Conjugated polymers bearing TEMPO units are successfully used as catalysts for the selective oxidation of primary alcohols. Moreover, such polymers should exhibit satisfactory capacitive properties for energy storage applications. Herein, a TEMPO-substituted EDOT-SNS-EDOT conjugated unit was electropolymerized, and the resulting polymer film was investigated for its electrochromic, capacitive, and electrocatalytic properties. The donor structure of the monomer facilitated efficient polymerization. The polymer film displayed brownish-pink and blue-gray colors in its neutral and oxidized states, respectively, while its semioxidized states showed brownish and khaki hues. The optical contrast was determined to be 28% at 505 nm, with oxidation and reduction times of 1.20 and 1.38 s, respectively. The areal-specific capacitance of the polymer electrode was calculated as 3.81 mF/cm^2^ (0.1 mA/cm^2^), ranking it at a high-mid level compared to other electrodeposited conjugated polymers reported in the literature. The electrocatalytic activity of the polymer film was evaluated using cyclic voltammetry (CV) in the presence of benzyl alcohol. The TEMPO units effectively catalyzed the oxidation of benzyl alcohol to benzaldehyde in the presence of 2,6-lutidine. Notably, the same polymer film was reused for three successive experiments without any loss of electrocatalytic activity. These findings demonstrate that the developed polymer film exhibits promising electrochromic, capacitive, and electrocatalytic properties.

## Graphical abstract

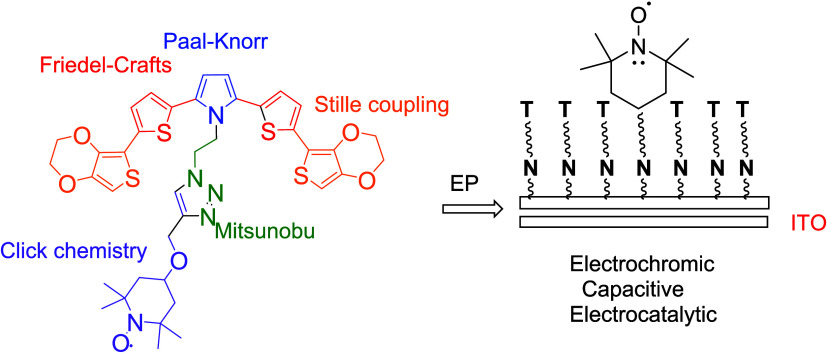

## Introduction

Nitroxides or nitroxyl radicals are characterized by an *N*,*N*-disubstituted N–O• unit, where the unpaired electron is delocalized within the nitrogen–oxygen bond.^1^ Among these, 2,2,6,6-tetramethylpiperidin-1-yloxy (TEMPO) and its derivatives have attracted significant attention over the past decades due to their low toxicity, high efficiency, and selectivity in oxidation reactions.^2–7^ TEMPO radicals serve as effective catalysts for oxidation reactions, enabling the conversion of alcohols into their corresponding aldehydes or ketones.^8,9^ However, catalyst recovery after the reaction remains a challenge. Recent approaches focus on immobilizing TEMPO onto solid supports such as silica,^10,11^ carbon nanotubes,^12,13^ graphene oxide,^14^ and polymers^10,11,15^ to address this issue. A particularly promising strategy involves TEMPO-substituted conjugated systems, which can be polymerized via electrochemical methods. The resulting polymer films function as “plug-and-play” electrodes, effectively eliminating the catalyst recovery problem from the reaction medium.^16–18^ Some conducting polymers, such as polythiophene^8,19–21^ and polypyrrole,^22,23^ are commonly used as supports for preparing TEMPO-modified electrodes.^24^ An ideal conducting polymer for this application should have a relatively low oxidation potential to ensure adequate electrochemical activity without suppressing TEMPO.^25^

Beyond their catalytic properties, TEMPO functionalized nonconductive and conductive polymers have also been explored as electrode materials for energy storage devices, exhibiting satisfactory charge storage and capacitive performance.^26–30^

Building on this information, a suitable TEMPO-anchored conjugated monomer was designed and synthesized. For instance, SNS, containing a central pyrrole ring with two external thiophenes, is a well-established conjugated unit with a relatively low oxidation potential.^31,32^ Furthermore, SNS can be readily functionalized via the central pyrrole ring.^33^ In this study, a TEMPO-substituted SNS unit was further modified by covalently bonding it to 3,4-ethylenedioxythiophene (EDOT) at both termini. The resulting monomer was expected to undergo facile polymerization, with the presence of EDOT enhancing both stability and optical properties.^34–36^ The polymer of the corresponding monomer was prepared by electrochemical polymerization, and the resulting polymer film was characterized for its fundamental electrochromic properties. To the best of our knowledge, this is the first study to investigate the electrochromic properties of a conjugated polymer containing TEMPO groups. In addition, its capacitive properties and electrocatalytic activity were examined.

## Results and Discussion

The synthetic route of the monomer is outlined below and illustrated in Scheme 1. A multistep strategy has been planned for the conjugated monomer to which the TEMPO unit will be anchored. The dual reaction site of 2-bromothiophene, is a suitable starting compound chosen both for the synthesis of the SNS core and for attaching the EDOT unit.

**Scheme 1:**
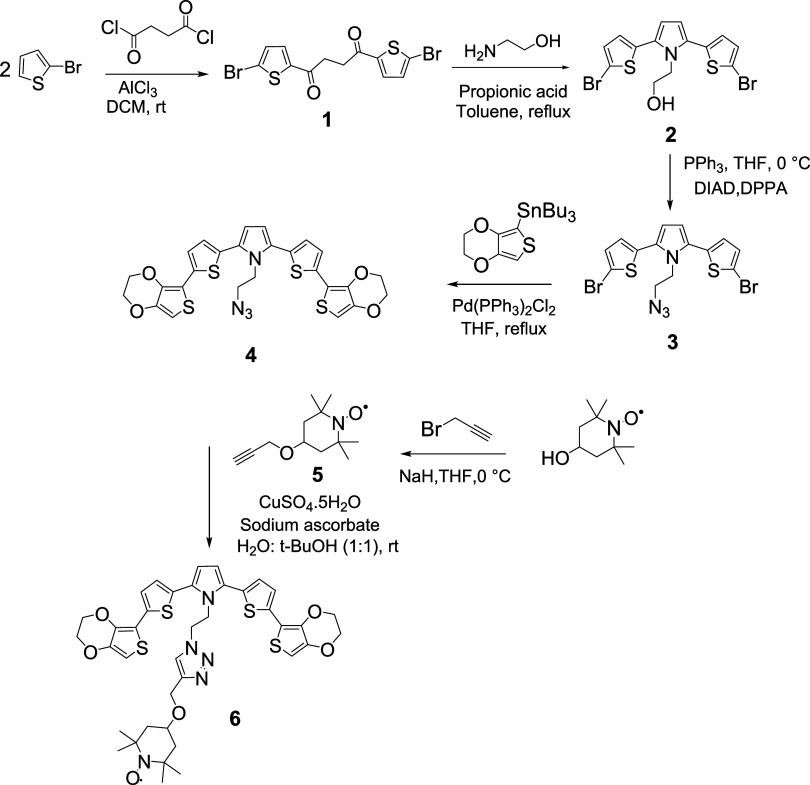
Synthesis of E-SN­(T)­S-E Monomer **6**

Through a Friedel–Crafts reaction with succinyl chloride in the presence of AlCl_3_, 1,4-bis­(5-bromothiophen-2-yl)­butane-1,4-dione (**1**) was synthesized.^37^ This intermediate underwent a Paal-Knorr type reaction with 2-aminoethan-1-ol in the presence of propionic acid as a catalyst to form the pyrrole motif of the SNS system.^38^ The resulting compound, 2-(2,5-bis­(5-bromothiophen-2-yl)-1H-pyrrol-1-yl)­ethan-1-ol (**2**), had its hydroxyl group converted into an azide group via a Mitsunobu-type reaction.^39^ Subsequently, compound **3** underwent a Stille reaction in the presence of a Pd catalyst with tributyl­(2,3-dihydrothieno­[3,4-*b*]­[1,4]­dioxin-5-yl)­stannane, yielding the 5-conjugated ESNSE-type donor motif **4**.^40^ The azide motif present in this structure serves as a suitable unit for click chemistry. Meanwhile, α-hydroxy TEMPO reacted with propargyl bromide in the presence of NaH, forming a triple bond-containing TEMPO compound **5**. In the final step, the desired TEMPO-anchored 5-conjugated ″E-SN­(T)­S-E″ monomer **6** was obtained via a click chemistry reaction.

E-SN­(T)­S-E monomer was characterized in terms of electrochemical behavior using cyclic voltammetry (CV) and differential pulse voltammetry (DPV). Electrochemical measurements were conducted using a platinum (Pt) disc as the working electrode, a Pt wire as the counter electrode, and an Ag/AgCl electrode as the reference electrode, respectively (Figure 1a,b). Oxidation of the monomer begins at approximately 0.55 V, with two distinct oxidation peaks observed at 0.63 and 0.77 V. The first oxidation peak is reversible and corresponds to a one electron transfer between TEMPO and TEMPO^+^ species,^41^ while the second peak is attributed to the oxidation of the E-SN­(T)­S-E monomer.

**Figure 1:**
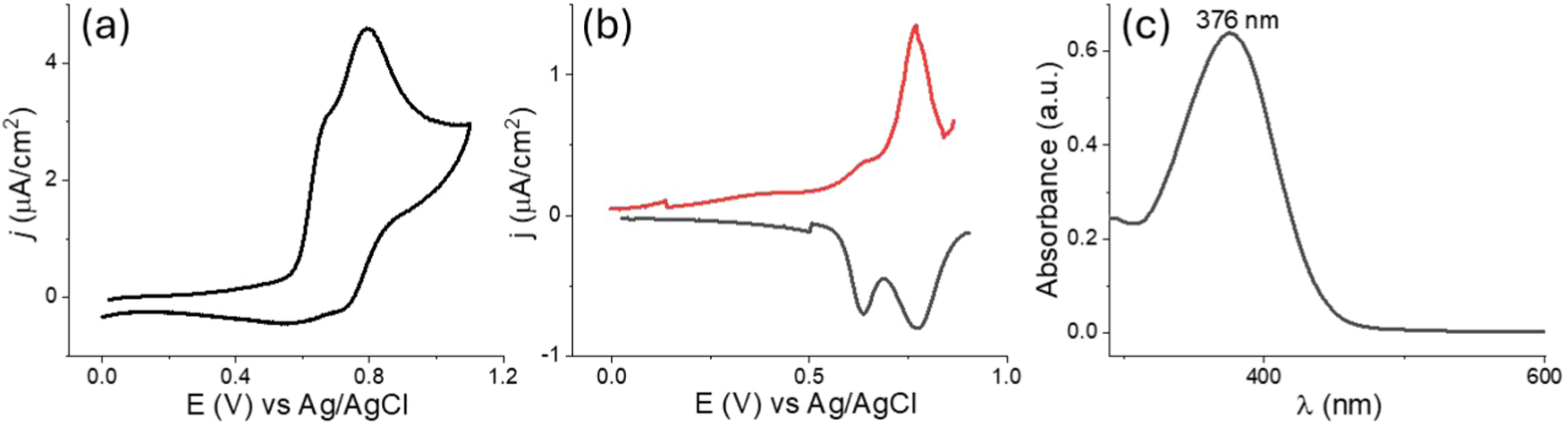
(a) CV (collected at a scan rate of 100 mV/s) and (b) DPV of E-SN­(T)­S-E monomer in DCM/TBAPF_6_ (Pt disc as the working electrode vs Ag/AgCl), (c) UV–vis spectrum of E-SN­(T)­S-E monomer in DCM.

The optical properties of the monomer were investigated by recording the UV–vis spectrum in DCM (Figure 1c). The maximum absorption wavelength (λ_max_) was determined to be 376 nm.

After characterizing the E-SN­(T)­S-E monomer, it was electropolymerized using cyclic voltammetry. The polymerization was carried out over 10 cycles, with the potential scanned between −0.50 and 1.05 V in a DCM/TBAPF_6_ electrolyte system on an indium tin oxide (ITO)-coated working electrode (Figure 2a). A progressive increase in current intensity was observed with each successive scan, indicating the formation of a polymeric film on the electrode surface. The electropolymerization reaction is depicted in Scheme 2.

**Figure 2:**
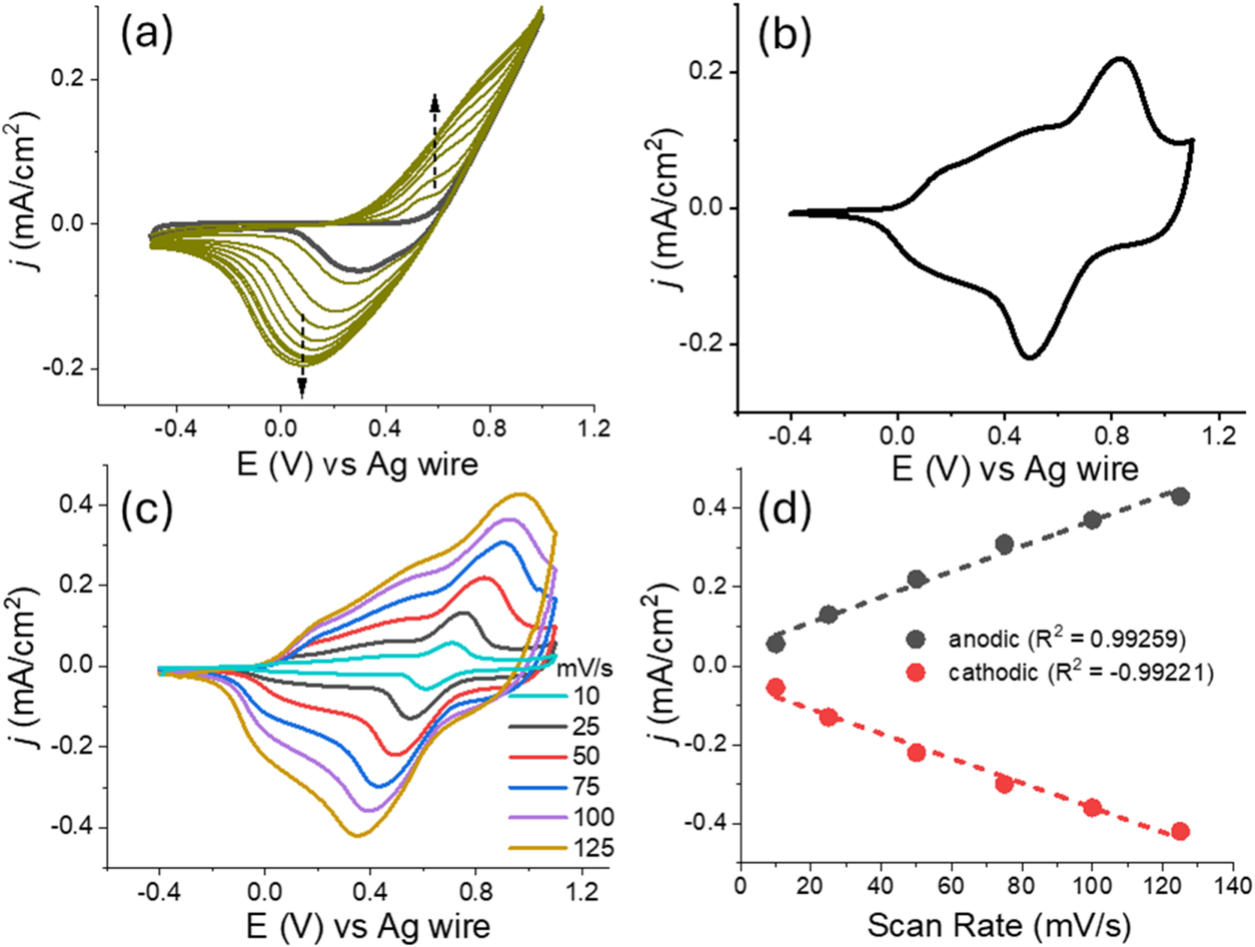
(a) The CVs of E-SN­(T)­S-E during electropolymerization process (recorded in DCM-TBAPF_6_,at a scan rate of 100 mV/s), (b) CV of the polymer film recorded between −0.5 and 1.1 V at a scan rate of 50 mV/s in ACN/TBAPF_6_, (c) CVs of the polymer film at increasing scan rates in ACN/TBAPF_6_, (d) anodic (black) and cathodic (red) current vs scan rate plots.

**Scheme 2:**
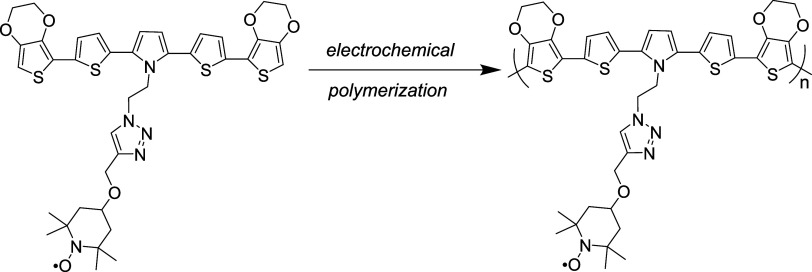
Electrochemical Polymerization of E-SN­(T)­S-E

After polymerization was completed, the brownish-pink colored polymer film was washed with acetonitrile (ACN) and transferred into monomer-free ACN/TBAPF_6_ electrolyte solution. The CV of the polymer film was then recorded at a scan rate of 50 mV/s. As shown in Figure 2b, a broad oxidation peak appeared between 0.4 and 0.6 V, along with a sharp oxidation peak around 0.8 V.

The CV behavior of the polymer film was further explored at scan rates ranging from 10 to 125 mV/s (Figure 2c). By plotting the anodic and cathodic current responses against the scan rate, a linear current–scan rate relationship was obtained (Figure 2d). This linear dependence confirms strong adhesion of the polymer film to the electrode surface and indicates that its redox behavior is not diffusion-controlled.^42^

Additionally, as reported previously,^26^ the existence of possible side reactions between TEMPO and the polymer backbone was also taken into consideration. It was evaluated that the presence of a flexible alkyl spacer between TEMPO and the polymer backbone significantly reduced charge transfer or radical-induced side reactions. Moreover, distinct redox peaks originating from both the polymer backbone and TEMPO (Figure 2b,2c) confirmed that the TEMPO units remained electrochemically active and intact after polymerization.

The UV–vis absorption behavior of the polymer film was monitored during oxidation from −0.5 to 1.0 V (Figure 3). The absorption maximum in the neutral state was located at 505 nm, and the optical band gap was determined as 1.88 eV from the onset of the neutral absorption band. Upon oxidation, the intensity of the neutral band began to decrease at approximately 0.11 V, while a new band centered around 800 nm emerged, likely due to polaron formation. At voltages exceeding 0.5 V, an additional absorption band emerged beyond 800 nm, suggesting the formation of bipolaronic bands. These spectral changes were naturally accompanied by distinct color transitions in the polymer film. In its neutral state, the polymer appeared brownish-pink (L:53.5, a:16.0, b:20.2). As oxidation progressed, the color transitioned to blue-gray (L:62.3, a:–3.5, b:8.8) in the fully oxidized state. In addition, brownish (L:73.1, a:7.3, b:24.4) and khaki (L:72.5, a:–1.8, b:19.8) hues were also observed in semioxidized states (Inset of Figure 3).

**Figure 3:**
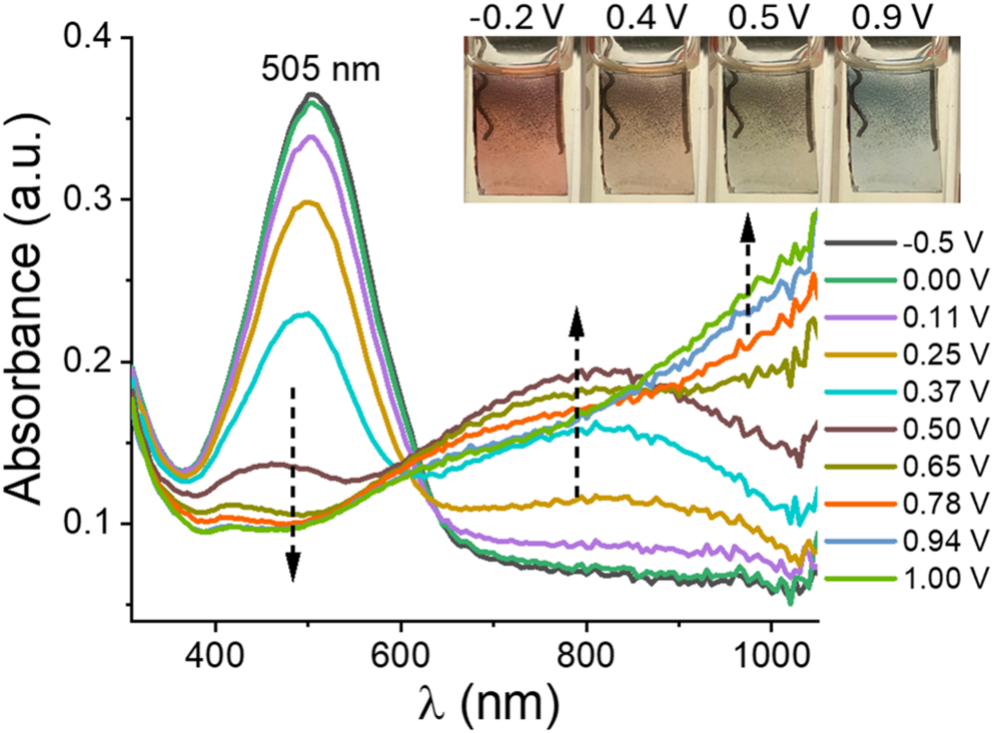
UV–vis spectra of the polymer film, monitored during the anodic scan from −0.5 to 1.0 V. Inset: Colors of the polymer film at its neutral and oxidized states.

A kinetic study was conducted on the polymer film to determine some key optical properties, including percent transmittance (T%) and switching times (t_ox_/t_red_). Constant potentials of −0.5 and 1.0 V were applied at 10 s intervals for reduction and oxidation, respectively (Figure S1a). Based on the 10th cycle of kinetic data, the percent transmittance was determined as 28% at 505 nm, with oxidation/reduction times of 1.20 s/1.38 s, respectively (calculated at 95% of the full optical contrast). The optical stability of the polymer film was tested over 50 oxidation/reduction cycles, retaining 71% of its optical contrast (Figure S1b). According to Schwartz et al., the incorporation of TEMPO units enhances the solubility of the conjugated system and facilitates the formation of a thin film on the electrode surface during electropolymerization.^43^ Additionally, the limited optical stability of the polymer film can be attributed to the gradual dissolution of the polymer material in the electrolyte over time.

While literature reports improved optical contrast and stability using alternative conjugated units or hybrid electrochromic systems,^44–46^ the electrochromic properties of TEMPO-functionalized SNS polymers have not been previously documented. For comparative purposes, the electrochromic characteristics of various N-functionalized SNS-based conjugated polymers are summarized in Table S1. Based on our findings, the integration of EDOT into the SNS backbone in this study led to enhanced optical performance. Notably, PEDOT itself exhibits an optical band gap of 1.65 eV and a switching time of 0.84 s (at 598 nm), and undergoes a color change from dark blue (L: 29, a: −0.4, b: 28) to transmissive sky blue (L:83, a: −8, b: −9).^42,49^ The lower optical band gap value and the faster response time of P­(E-SN­(T)­S-E), compared to other SNS polymers listed in Table S1, further confirm the positive influence of the EDOT unit on the optical properties of the resulting polymer.

The capacitive behavior of the polymer film was also investigated using chronopotentiometry technique. Areal-specific capacitance values (mF/cm^2^) were calculated from the galvanostatic charge–discharge (GCD) measurements, performed at varying currents, using the following equations

1 C=[j×Δt]/ΔV

where *C* is the areal-specific capacitance, *j* is the current density (per cm^2^), Δ*t* is the discharge time, and Δ*V* is the potential window for discharge.^42,47^

Figure 4 illustrates the results of capacitive study. The GCD curves of the polymer exhibit symmetrical responses during the charging/discharging process. Furthermore, these symmetrical GCD shapes are maintained even at higher current values, indicating a satisfactory charge/discharge rate capability.^48,49^ The areal-specific capacitance of the polymer film was calculated as 3.81 mF/cm^2^ (at 0.1 mA), placing it at a high-mid level compared to reported electrodeposited electrochromic polymers (Table 1).^42,49–53^

**Figure 4:**
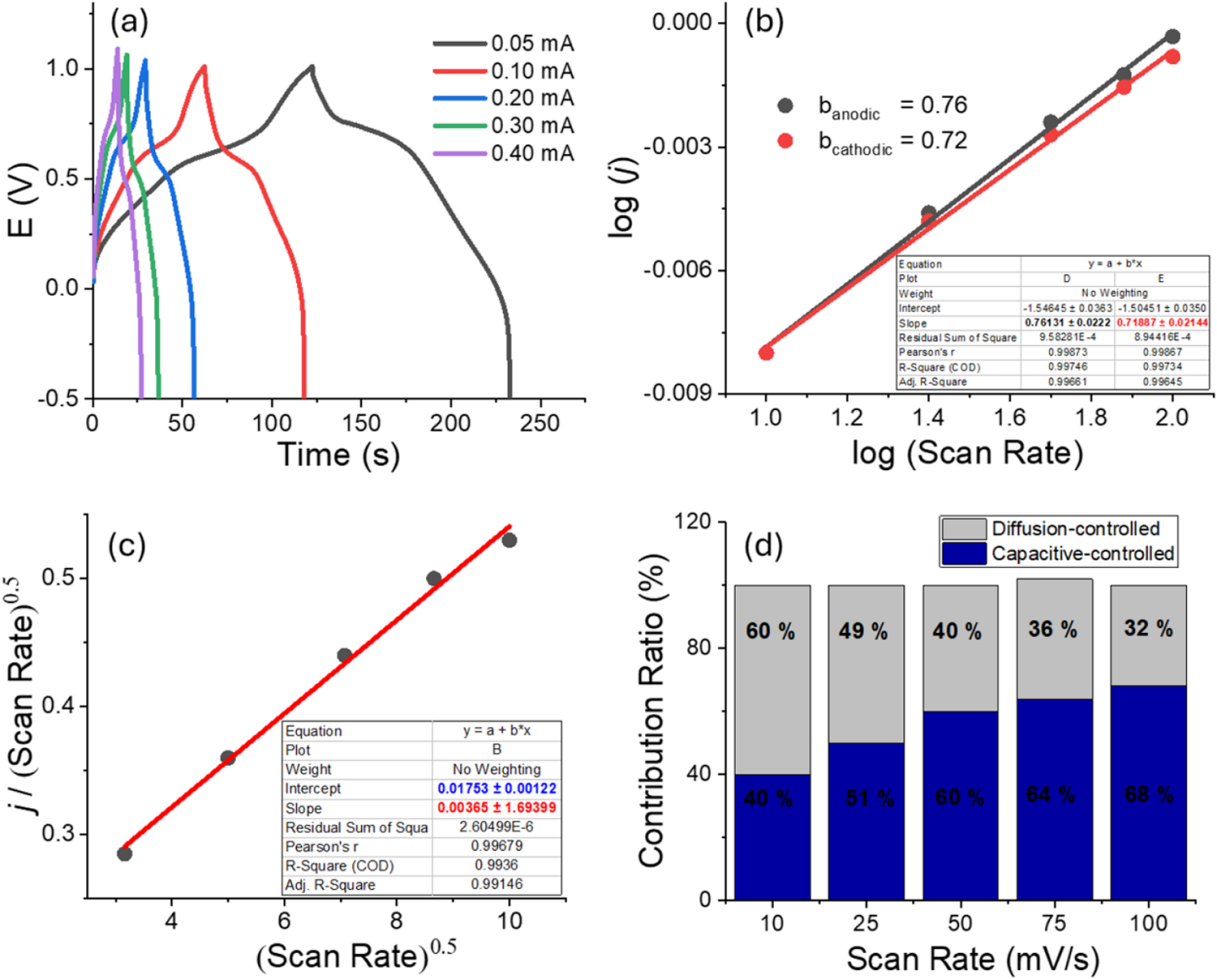
(a) GCD curves of polymer film at varying currents, (b) b-value plot of the polymer film both for anodic and cathodic currents, (c) the graph for obtaining the slope and intercept which is used for capacitive contribution calculations, (d) capacitance contribution ratio diagram of the polymer film at varying scan rates.

**Table 1:** Areal Specific Capacitance of Electrodeposited Polymer Films: A Literature Comparison

polymer synthesis	C (mF/cm^2^)	current density (mA/cm^2^)	ref
electrodeposition	3.81	0.10	this work
electrodeposition	1.58	0.01	^42^
electrodeposition	2.47	0.05	^43^
electrodeposition	2.58	0.05	^44^
electrodeposition^a^	3.00	0.10	^41^
electrodeposition^a^	4.07	0.044	^38^
electrodeposition	4.65	0.05	^45^

a Refers to electrochemical copolymerization

The capacitive stability was also tested by subjecting the polymer to 50 repetitive GCD-cycles (Figure S2). The capacitance retention rate was then calculated using the following equation:

2 $$C\%=\left(C_{50}/C_{1}\right)\;\times \;100$$

where *C* % is the capacitance retention rate, *C*_50_ is the specific capacitance of 50th cycle, and *C*_1_ is the initial specific capacitance.

Gratifyingly, the capacitance retention rate after 50 GCD cycles was calculated to be 93%, suggesting that the polymer film could be a suitable candidate for use as an electrode material in electrochemical storage applications (Figure S2).^54^ When the capacity retention of the P­(E-SN­(T)­S-E) electrode is compared with other TEMPO-bearing conjugated polymers reported in the literature (typically used as composites in battery applications and tested over 50,^55,56^ 60,^57^ and 200 ^58^ GCD cycles), the bare electrode presented in this study demonstrates a satisfactory and competitive capacitive performance.^55–58^

To determine whether the capacitive behavior of the polymer film is dominated by capacitive or diffusion controlled, the relationship between the peak current and the scan rate of the redox process was investigated (Figure 4b).^42^ The slope of the corresponding plot gives the b value. When the b-value approaches to 1.0, the system is primarily controlled by capacitance rather than diffusion.^59^ The b values obtained for the anodic and cathodic peaks were found to be 0.76 and 0.72, respectively, indicating contributions from both capacitance and diffusion. The capacitance contribution ratios were calculated at varying scan rates using Dunn’s method:

The current values were measured for a selected potential at each scan rate. Dunn’s method uses the formula

3 $$i\left(V\right)=k_{1}\nu +k_{2}\nu^{1/2}$$

Where *i*(*V*) is the current at a given voltage, ν is the scan rate, *k*_1_ν is the capacitive current (surface-controlled) and *k*_2_ν^1/2^ is the diffusion-controlled current.

To obtain *k*_1_ and *k*_2_, the formula is rearranged as follows

4 $$i\left(V\right)/\nu^{1/2}=k_{1}\nu^{1/2}+k_{2}$$

Plotting (*i*(*V*)/ν^1/2^) vs ν^1/2^ graph gives the slope as *k*_1_ and the intercept as *k*_2_ (Figure 4c).

Then the following equation calculates the % capacitive contribution at each scan rate (Figure 4d)

$$\%\mathrm{capacitive}-\mathrm{ratio}=\left(k_{1}\nu \right)/\left(k_{2}\nu^{1/2}+k_{1}\nu \right)\;\times \;100$$

As is shown in Figure 4d, the capacitance contribution ratios were found to be 51% (at 25 mV/s) and 68% (at 100 mV/s). As seen from the results, the capacitive-controlled redox process becomes more dominant with increasing scan rates, as the diffusion rate of electrolyte ions is unable to compete with the charge storage rate, leading to enhanced surface capacitance control.^42,51^

The pendant TEMPO unit can be used as a catalyst in the oxidation of primary alcohols to the corresponding aldehydes.^28^ Numerous conducting polymer films bearing TEMPO radicals have been reported in the literature, where they were investigated as plug-and-play electrodes for the electro-oxidation of alcohols.^3,8,10,16,19,20,22,24,25,29^ This approach offers the advantage of easy recovery of TEMPO unit at the end of the catalytic oxidation reaction, as it is immobilized onto the polymer chain.

The electrocatalytic activity of the polymer film in the oxidation of benzyl alcohol (BA) substrate (in the presence of 2,6-lutidine (LUT) as a base) was investigated in ACN/TBAPF_6_. The electrochemical response of P­(E-SN­(T)­S-E) was monitored during oxidation using cyclic voltammetry (Figure 5). The addition of BA into the solution did not affect the redox behavior of the polymer. However, when LUT was introduced into the solution, the anodic peak current increased significantly, most probably due to the reaction between TEMPO radicals and benzyl alcohol, mediated by LUT. The formation of benzaldehyde—the oxidation product of the electrocatalytic reaction was monitored using thin-layer chromatography (TLC) (Figure S3).

**Figure 5:**
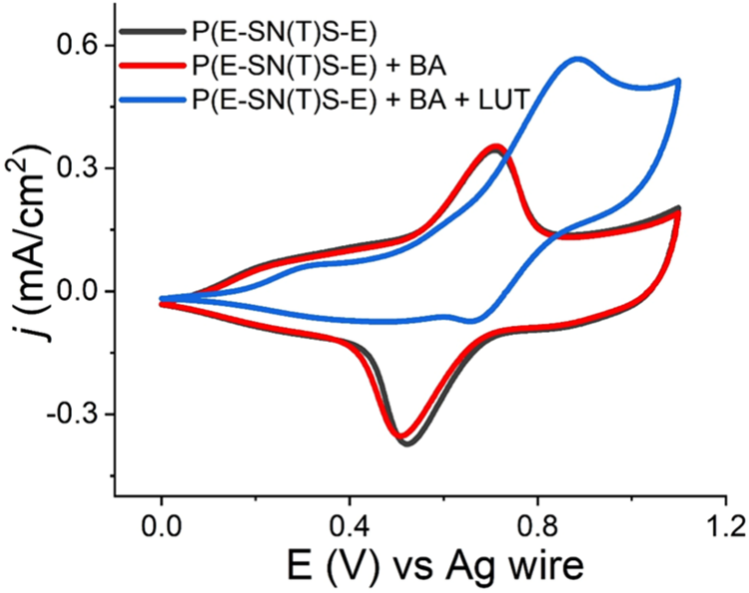
Cyclic voltammogram of P­(E-SN­(T)­S-E) in the presence of BA (red line) and in the presence of BA+LUT (10 mmol:10 mmol).

The same electrode was then used in the two successive alcohol-oxidation runs after being washed with ACN. Gratifyingly, it was observed that the electrode remained active as the electro-catalyst in the oxidation of benzyl alcohol (Figure S4). The proposed mechanism is shown in Scheme 3.

**Scheme 3:**
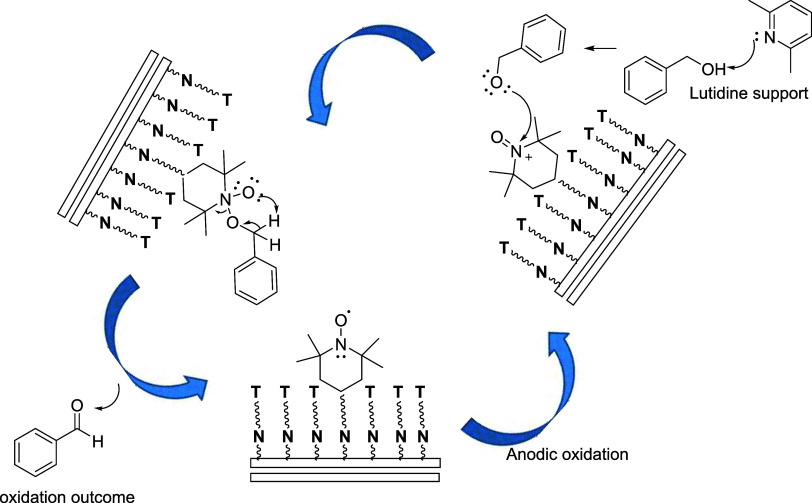
Proposed Mechanism of Oxidation Reaction

## Conclusions

In conclusion, a TEMPO-incorporated EDOT-SNS-EDOT monomer has been synthesized and electropolymerized, and the resulting polymer film was investigated for its electrochromic, capacitive, and electrocatalytic activity properties. Donor nature of the monomer supplied facilitated easy polymerization. The resulting polymer film exhibited brownish-pink and blue-gray colors in its neutral and oxidized states, respectively. In addition, brownish and khaki colors were observed at semioxidized states. The optical contrast was calculated to be 28% (at 505 nm) with the oxidation/reduction times were calculated as 1.20 s/1.38 s, respectively.

The areal-specific capacitance of the polymeric electrode was determined from GCD measurements and found to be 3.81 mF/cm^2^, placing it in the high-mid level range compared to previously reported electrodeposited conjugated polymers in the literature.

The electrocatalytic activity of the polymer film was monitored using CV in the presence of benzyl alcohol with TEMPO units serving as the catalytic sites for the oxidation of benzyl alcohol to benzaldehyde. The polymer film was reused in three successive oxidation experiments and retained its electrocatalytic activity. These results demonstrate that the polymer film developed herein exhibits excellent electrochromic, capacitive, and electrocatalytic properties.

## Experimental Section

### General Information

All materials were obtained from commercial sources. Solvents were distilled prior to use. Reagents and solvents (tetrabutylammonium hexafluorophosphate (TBAPF_6_), 3,4-ethylenedioxy thiophene (EDOT), acetonitrile (ACN), ethyl acetate (EtOAc) and dichloromethane (DCM)) were purchased from Merck or Sigma-Aldrich. The electrochemical and spectroelectrochemical measurements were performed using Gamry PCI4/300 potentiostat-galvanostat and Carry 60 model UV–vis spectrometer combined with Gamry, respectively. Electrochemical measurements for the monomer were conducted using a platinum (Pt) disc as the working electrode, a Pt wire as the counter electrode, and an Ag/AgCl electrode as the reference electrode, respectively. The polymers were deposited on ITO (Delta Tech. 8–12, 0.7 cm × 5 cm) coated glass working electrode (vs Ag wire as pseudoreference, and platinum wire as counter electrodes). All polymerizations were conducted in DCM/TBAPF_6_ electrolyte system, and the polymer behaviors were investigated in ACN/TBAPF_6_. The color values of the polymer films (L: brightness; a: hue, b: saturation) were reported according to “The International Commission of Illumination, CIE,” color coordinates. ^1^H and ^13^C NMR spectra used in the characterization of the materials were obtained using a Bruker Spectrospin Avance DPX-400 Spectrometer in CDCl_3_ solvent. During the measurements, chemical shifts were calculated with respect to tetramethylsilane (TMS) as the internal standard. The data obtained in ppm units were given with the abbreviations s (singlet), bs (broad singlet), d (doublet), dd (doublet of doublet), ddd (doublet of doublet of doublet), dtt (doublet of triplet of triplet), t (triplet), td (triplet of doublet), m (multiplet). Coupling constants (J) were given in Hertz (Hz). Bruker α Platinum ATR was used to record infrared spectroscopy results. HRMS data were recorded with Agilent 6224 TOF LC/MS. The melting points of the compounds were obtained with MEL-TEMP 1002D.

### Experimental Procedures

^1^H and ^13^C NMR spectra for all the compounds are provided in Supporting Information file.

#### Synthesis of 1,4-bis­(5-Bromothiophen-2-yl)­butane-1,4-dione **(1)**

A solution of 2-bromo thiophene (2.4 mL, 0.03 mol) in dichloromethane (DCM) (7.5 mL) and succinyl chloride (1.65 mL, 0.015 mol) was prepared. This solution was then added dropwise to a stirring mixture of AlCl_3_ (4 g, 0.03 mol) in DCM (3.75 mL) at room temperature. The resulting mixture was stirred for 20 h under an argon (Ar) atmosphere. Subsequently, a mixture of chrushed ice (22.5 g) and concentrated hydrochloric acid (HCl, 2.5 mL) was added to the reaction medium and stirred for an additional 2 h. The organic phase was extracted sequentially with 10% sodium bicarbonate (NaHCO_3_) and 10% sodium chloride solutions, respectively. The extract was then dried over anhydrous Na_2_SO_4_. After solvent removal under reduced pressure, the crude product was purified by recrystallization from ethanol. Following filtration, compound **1** was obtained as blue-silver crystals in a 63% yield. mp: 177–178 °C. ^1^H NMR (400 MHz, Chloroform-*d*): δ 7.54 (d, *J* = 4.0 Hz, 2H), 7.12 (d, *J* = 3.9 Hz, 2H), 3.29 (s, 4H) ppm. ^13^C NMR (101 MHz, CDCl_3_): δ 188.1, 143.0, 131.3, 130.3, 121.9, 31.4 ppm. IR (neat): 2902, 1651, 1525, 1417, 1329, 1211, 1190, 1071, 985, 942, 908, 814, 785, 735, 711 cm^–1^.

#### Synthesis of 2-(2,5-bis­(5-Bromothiophen-2-yl)-1H-pyrrol-1-yl)­ethan-1-ol **(2)**

A two-necked round-bottom flask was charged with 1,4-bis­(5-bromothiophen-2-yl)­butane-1,4-dione (**1**) (1.24 g, 3.00 mmol), excess ethanolamine (0.9 mL, 15.00 mmol) and a catalytic amount of propionic acid (0.04 mL, 0.50 mmol) in anhydrous toluene (30 mL). The reaction mixture was heated under reflux at 110 °C for 16 h using a Dean–Stark trap apparatus. After completion, the reaction was cooled to room temperature, and the solvent was removed under reduced pressure. The crude product was purified by silica gel column chromatography with a 1:5 EtOAc:hexanes eluent, giving compound **2** as a yellow solid (0.968 g, 75% yield). mp: 62–63 °C. ^1^H NMR (400 MHz, Chloroform-*d*): δ 7.03 (d, *J* = 3.8 Hz, 2H), 6.88 (d, *J* = 3.8 Hz, 2H), 6.31 (s, 2H), 4.28 (t, *J* = 6.0 Hz, 2H), 3.66 (t, *J* = 6.1 Hz, 2H) ppm. ^13^C NMR (101 MHz, CDCl_3_): δ 135.9, 130.4, 128.1, 127.2, 112.2, 111.9, 62.0, 46.6 ppm. IR (neat): 3295, 3120,3101, 2978, 2947, 2926, 2894, 2882, 2850, 1651, 1573, 1457, 1402, 1325, 1187, 1042, 962, 789, 766 cm^–1^. HRMS (ESI) *m*/*z* calculated for C_14_H_13_Br_2_NOS_2_ [M + H]^+^ 431.87216, found 431.87296.

#### 1-(2-Azidoethyl)-2,5-bis­(5-bromothiophen-2-yl)-1H-pyrrole (**3**)

A solution of 2-(2,5-bis­(5-bromothiophen-2-yl)-1H-pyrrol-1-yl)­ethan-1-ol (**2**) (866.4 mg, 2 mmol) and triphenylphosphine (629.5 mg, 2.4 mmol) was prepared in anhydrous tetrahydrofuran (THF) (10 mL) at 0 °C. Subsequently, diisopropyl azodicarboxylate (DIAD) (0.47 mL, 2.4 mmol) was added in one portion. In a separate flask, diphenyl phosphoryl azide (DPPA) (2.58 mL, 12 mmol) was dissolved in 20 mL anhydrous THF. The DPPA solution was then added dropwise to the reaction mixture at 0 °C. Following the addition, the reaction mixture was gradually warmed to room temperature and further stirred for 12 h. The crude product was purified, affording compound **3** as a yellow oil in a 70% yield. ^1^H NMR (400 MHz, Chloroform-*d*): δ 7.05 (d, *J* = 3.8 Hz, 2H), 6.86 (d, *J* = 3.8 Hz, 2H), 6.34 (s, 1H), 4.30 (t, *J* = 6.3 Hz, 2H), 3.29 (t, *J* = 6.3 Hz, 2H) ppm. ^13^C NMR (101 MHz, CDCl_3_): δ 135.6, 130.5, 128.0, 127.2, 112.5, 112.4, 50.9, 44.1 ppm. IR (neat): 3113, 2985, 2923, 2870, 2127, 2098, 1513, 1484, 1432, 1362, 1160, 1059, 1014, 968, 909, 881, 807, 771, 708 cm^–1^. HRMS (ESI) *m*/*z* calculated C_14_H_10_Br_2_N_4_S_2_ [M + H]^+^ 456.87864, found 456.87944.

#### 1-(2-Azidoethyl)-2,5-bis­(5-(2,3-dihydrothieno­[3,4-*b*]­[1,4]­dioxin-5-yl)­thiophen-2-yl)-1H-pyrrole (**4**)

An anhydrous THF (50 mL) solution of 1-(2-azidoethyl)-2,5-bis­(5-bromothiophen-2-yl)-1H-pyrrole (**3**) (0.50 g, 1.09 mmol) and 2-(tributyltin)-3,4-ethylenedioxythiophene (1.18 g, 2.73 mmol) was prepared. Bis­(triphenylphosphine)­palladium­(II) dichloride (0.172 g, 0.245 mmol) was added at room temperature under a nitrogen atmosphere. The reaction mixture was heated under reflux for 48 h. After completion, the reaction was concentrated under reduced pressure, diluted with water, and extracted with EtOAc. The organic phase was washed with brine, dried over anhydrous Na_2_SO_4_, and concentrated under reduced pressure. The crude product was purified by silica gel column chromatography using a 1:3 EtOAc:hexanes eluent, giving compound **4** as a yellow solid (0.215 g, 34% yield). mp: 110 °C (decomp.). ^1^H NMR (400 MHz, Chloroform-*d*): δ 7.19 (dd, *J* = 3.8, 1.0 Hz, 2H), 7.00 (d, *J* = 3.7 Hz, 2H), 6.40 (s, 2H), 6.25 (s, 2H), 4.44 (t, *J* = 6.5 Hz, 2H), 4.38–4.33 (m, 4H), 4.28–4.24 (m, 4H), 3.33 (t, *J* = 6.4 Hz, 2H) ppm. ^13^C NMR (101 MHz, CDCl_3_): δ 140.9, 136.7, 134.1, 131.3, 127.8, 125.2, 121.9, 110.9, 110.9, 96.1, 64.0, 63.6, 49.9, 43.3 ppm. IR (neat): 3114, 3102, 2925, 2870, 2097, 1648, 1514, 1484, 1431, 1362, 1297, 1160, 1059, 1014, 968, 908, 881, 830, 807, 771, 707 cm^–1^.

#### Synthesis of 4-Propargyloxy-TEMPO (**5**)

4- hydroxy TEMPO (1 g, 5.8 mmol) was added portionwise to a solution of sodium hydride (NaH, 279 mg, 6.97 mmol, 60% in mineral oil) in in anhydrous DMF (3.5 mL) at 0 °C. The reaction mixture was stirred for 1 h, after which propargyl bromide (0.6 mL, 6.97 mmol) was added dropwise at 0 °C. The reaction progress was monitored by thin-layer chromatography (TLC), and upon complete consumption of the starting material, the reaction mixture was quenched with ice-cold water (20 mL). The resulting mixture was extracted with EtOAc washed with brine, and dred over anhydrous MgSO_4_. The solvent was removed under reduced pressure, and the residue was purified by column chromatography. Compound **5** was obtained as an orange solid in a 47% yield. The NMR data confirmed the absence of phenylhydrazine signals that appeared in the ^1^H NMR. mp: 51–53 °C. ^1^H NMR (400 MHz, Chloroform-*d*): δ 4.09 (d, *J* = 2.5 Hz, 2H), 3.84–3.71 (m, 1H), 2.36 (t, *J* = 2.4 Hz, 1H), 1.90 (ddd, *J* = 11.2, 4.1, 1.8 Hz, 2H), 1.45 (t, *J* = 11.9 Hz, 2H), 1.17 (s, 6H), 1.12 (s, 6H) ppm. ^13^C NMR (101 MHz, CDCl3): δ 151.3 (phenylhydrazine), 129.3 (phenylhydrazine), 128.4 (phenylhydrazine), 119.5 (phenylhydrazine), 112.2 (phenylhydrazine), 80.1, 74.3, 69.7, 60.1, 55.4, 44.1, 31.7, 20.9 ppm.

#### Synthesis of **E-SN­(T)­S-E** Monomer (**6**)

Sodium ascorbate (0.991 g 0.5 mmol) and copper­(II) sulfate pentahydrate (CuSO_4_·5H_2_O, 0.125 g, 0.5 mmol) were added to a solution of substituted azide **4** (0.105 g, 0.55 mmol) and alkyne **5** (0.105 g, 0.5 mmol) in *tert*-butanol/water mixture (*t*-BuOH:H_2_O, 3 mL:3 mL). The reaction mixture was stirred at 40 °C until complete consumption of the starting materials. The reaction was then diluted with diethyl ether, extracted with distilled water, and dried over anhydrous Na_2_SO_4_. The solvent was removed under reduced pressure, and the crude product was purified by silica gel column chromatography using EtOAc as the eluent affording E-SNS-T monomer (0.127 g, 32% yield). ^1^H NMR (400 MHz, Chloroform-*d*): δ 7.14 (d, *J* = 3.6 Hz, 1H), 6.82 (s, 1H), 6.77 (d, *J* = 3.7 Hz, 1H), 6.36 (s, 1H), 6.24 (s, 1H), 4.71 (t, *J* = 6.1 Hz, 2H), 4.55 (s, 2H), 4.44 (t, *J* = 6.1 Hz, 2H), 4.36 (dd, *J* = 5.6, 2.6 Hz, 4H), 4.29–4.23 (m, 4H), 3.84 (s, 1H), 1.96 (d, *J* = 13.4 Hz, 2H), 1.75 (d, *J* = 4.5 Hz, 2H), 1.68 (s, 6H), 1.51 (s, 6H) ppm. ^13^C NMR (101 MHz, CDCl_3_): δ 151.5, 140.9, 135.5, 134.4, 130.4, 128.0, 127.9, 127.5, 125.2, 122.1, 99.0, 69.6, 64.2, 63.6, 59.4, 56.6, 39.5, 28.7, 25.9, 21.6 ppm. IR (neat): 3102, 2961, 2925, 2869, 2757, 2486, 2466, 1733, 1717, 1700, 1652, 1518, 1487, 1437, 1364, 1260, 1228, 1166, 1066, 1016, 969, 908, 883, 798, 732, 701 cm^–1^. HRMS (ESI) *m*/*z* calculated for C_38_H_42_N_5_O_5_S_4_^+^ [M-O]^+^ 776.2063, found 776.2022.

## Supplementary Material



## Data Availability

The data underlying this study are available in the published article and its online Supporting Information.

## Notes

Experimental details, characterization data, copies of NMR spectra (PDF)
