# Supplementary material for: Electrochromic, Capacitive, and Electrocatalytic Performance of TEMPO Anchored EDOT-SN(T)S-EDOT Electrode
Source: ACS Omega. 2025 Jul 3;10(27):29442–51. doi: 10.1021/acsomega.5c02762 (PMC12268454; doi:10.1021/acsomega.5c02762)
Supplement: Supplementary file 1 [file ao5c02762_si_001.pdf]

Supporting Information

for

Electrochromic, Capacitive, and Electrocatalytic Performance of TEMPO Anchored EDOT-SN(T)S-EDOT Electrode

Begum Nemutlu[a], Emre Fatih Eker[a], Ahmet M. Önal[a], Emine Gul Cansu Ergun,[b]*Cihangir Tanyeli[a]*

[a] *Department of Chemistry, Middle East Technical University, 06800 Ankara, Türkiye.*

E-mail: tanyeli@metu.edu.tr

[b] *Baskent University, Department of Electrical and Electronics Engineering, Ankara, Türkiye.*

E-mail: egulcansu@baskent.edu.tr

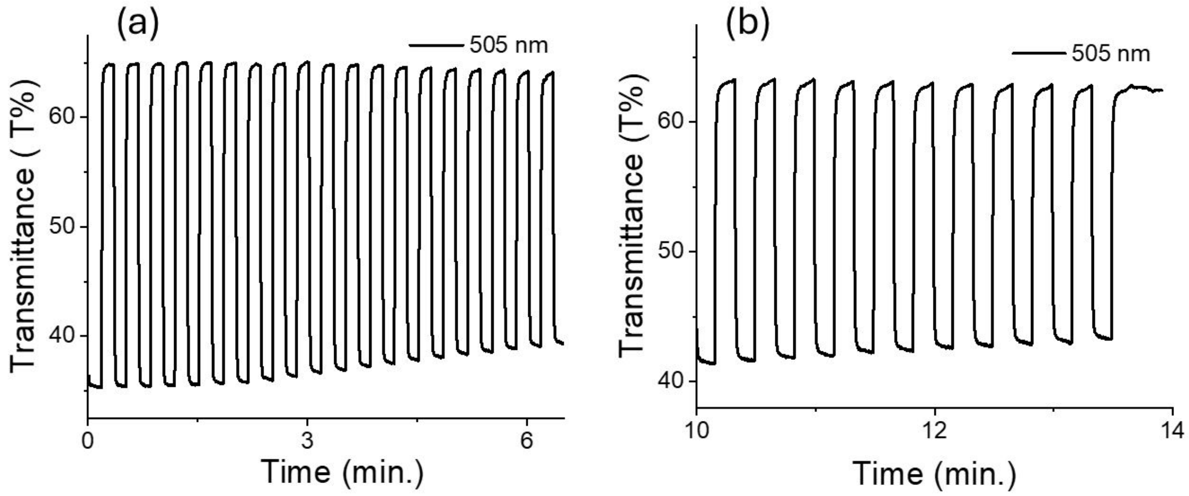

Figure S1. Chronoabsorptometry experiments of the polymer film, under constant potentials of -0.5 V and 1.0 V with 10 s intervals, **a)** The first 20 cycles, **b)** last 10 cycles of 50 cycles.

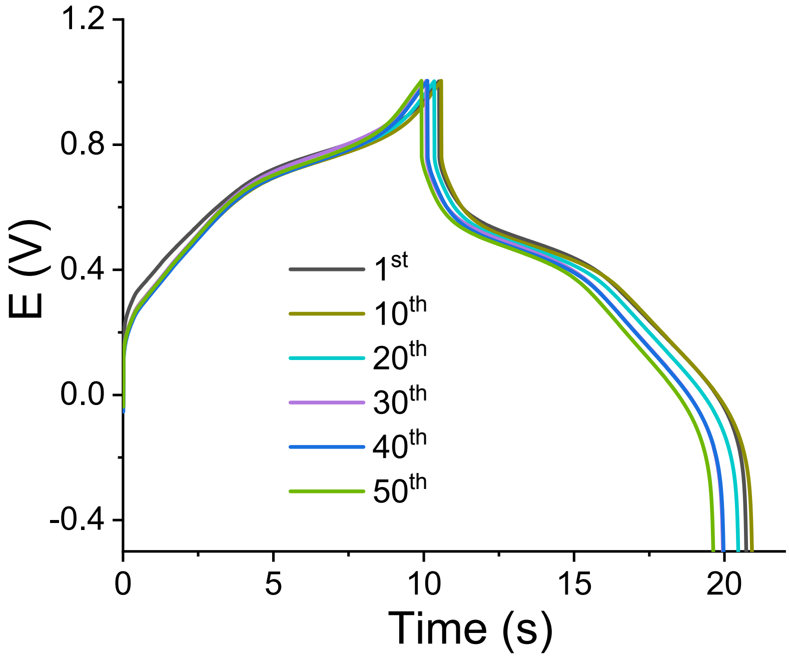

Figure S2. GCD curves of the polymer film during 50-GCD cycles (at 0.4 mA/cm^2 current density).

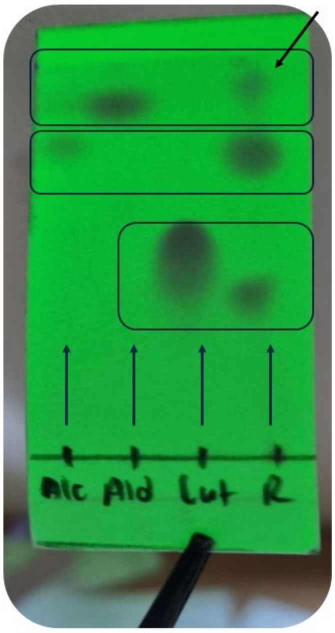

Figure S3. TLC spots of (from left to right): BA, aldehyde, LUT and the reaction mixture.

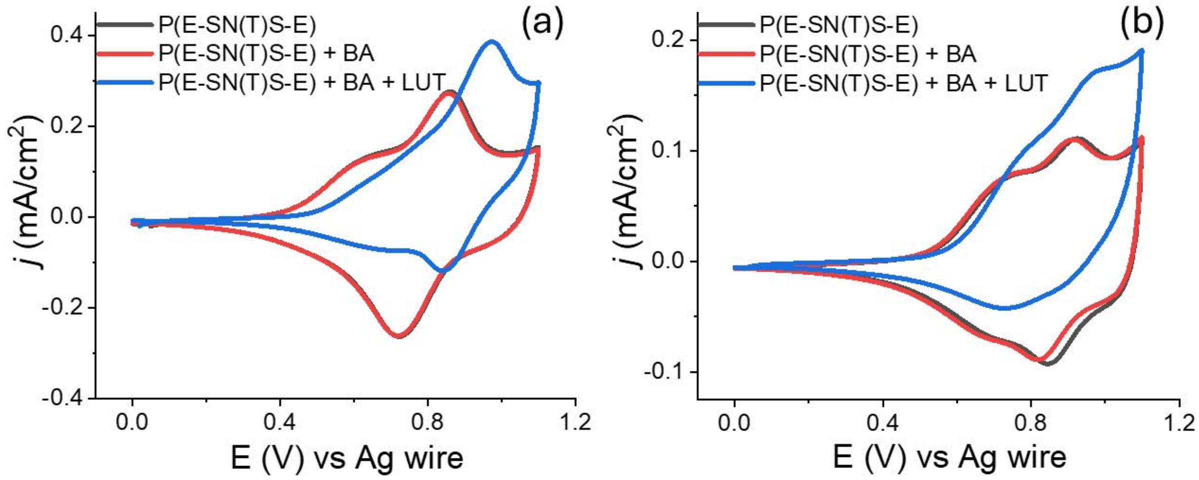

Figure S4. Electrocatalytic experiments of P(E-SN(T)S-E). Cyclic voltammogram of P(E-SN(T)S-E) in the presence of BA (red line) and in the presence of BA+LUT (10 mmol:10 mmol). (**a** and **b** demonstrate the repetitive experiments for the same polymer electrode.)

Table S1. Electrochromic properties of SNS-based electrodeposited polymer films: a literature comparison.

**Polymer**	**$E_{g(optical)}$ (eV)**	**ΔT (%)**	**t_{ox} (s)**
This work	1.88	28 (505 nm)	1.20
[1]	2.15	29.5 (435 nm)	2.5
[2]	1.95	18 (440 nm)	3.0
[3]	1.99	23 (430 nm)	2.5
[4]	2.20	18 (780 nm)	4.0
[5]	2.61	24 (830 nm)	2.9

References:

(1) Gumusay, O.; Soganci, T.; Durur, S.; Soyleyici, H. C.; Cetisli, H.; Ak, M. Purpald containing poly(2,5-dithienylpyrrole)-based multifunctional conducting polymer: synthesis, characterization, and electrochromic properties. *Ionics* **2020**, *26*, 3501-3511.

(2) Soyleyici, H. C.; Ak, M.; Şahin, Y.; Demikol, D. O.; Timur, S. New class of 2,5-di(2-thienyl)pyrrole compounds and novel optical properties of its conducting polymer. *Mater. Chem. Phys*. **2013**, *142*, 303–310.

(3) Soganci, T.; Ak, M.; Giziroglu, E.; Soyleyici, H.C. Smart window application of a new hydrazide type SNS derivative. *RSC Adv*. **2016**, *6*, 1744–1749.

(4) Wang, G.; Fu, X.; Huang, J.; Wu, L.; Du, Q. Synthesis and spectroelectrochemical properties of two new dithienylpyrroles bearing anthraquinone units and their polymer films. *Electrochim. Acta* **2010**, *55*, 6933–6940.

(5) Cai, S.; Wen, H.; Wang, S.; Niu, H.; Wang, C.; Jiang, X.; Bai, X.; Wang, W. Electrochromic polymers electrochemically polymerized from 2, 5-dithienylpyrrole (DTP) with different triarylamine units: Synthesis, characterization and optoelectrochemical properties. *Electrochim, Acta* **2017**, *228*, 332–342.

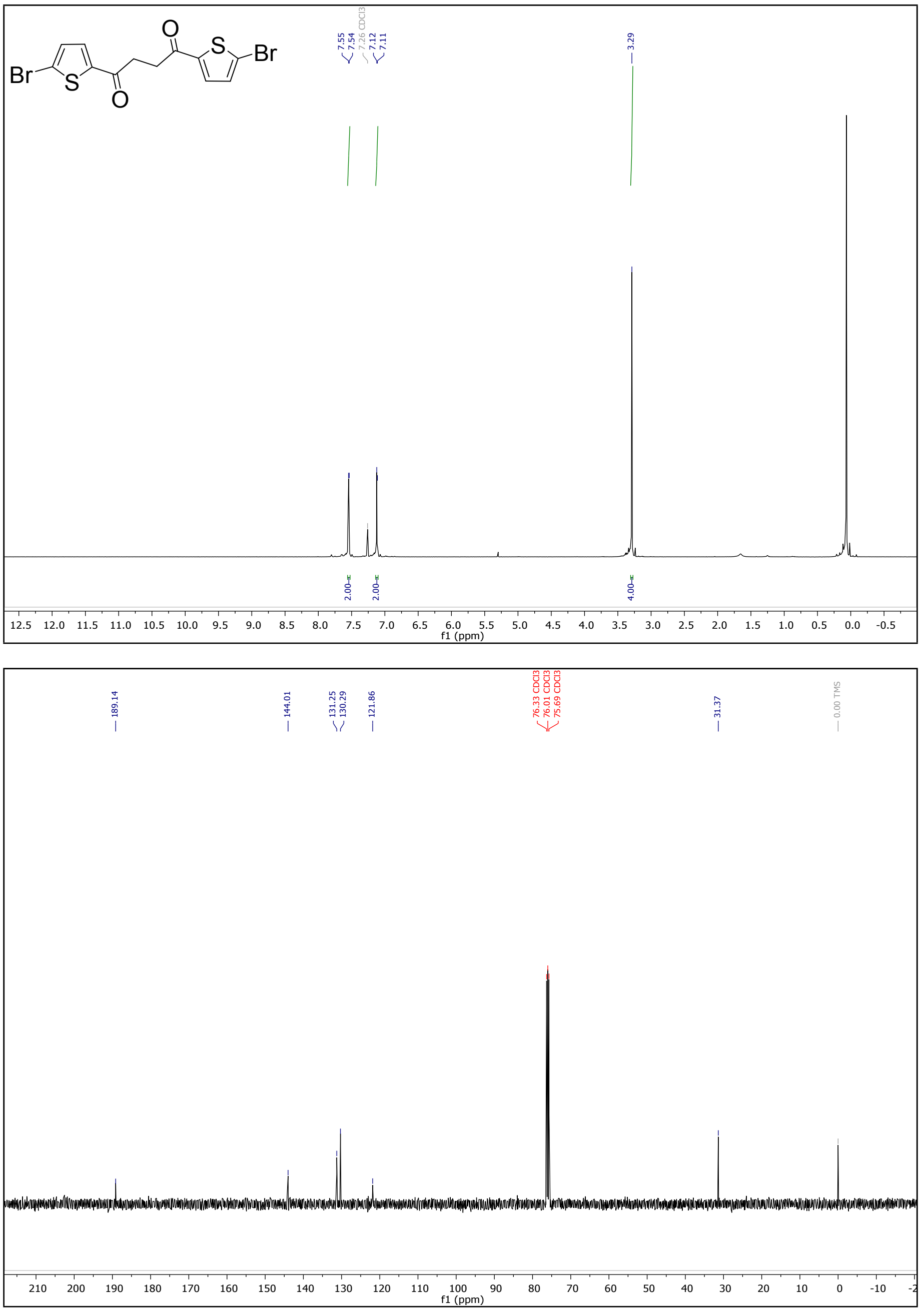

Figure S5. ^{1}H and ^{13}C NMR Spectra of 1,4-Bis(5-bromothiophen-2-yl)butane-1,4-dione (1)

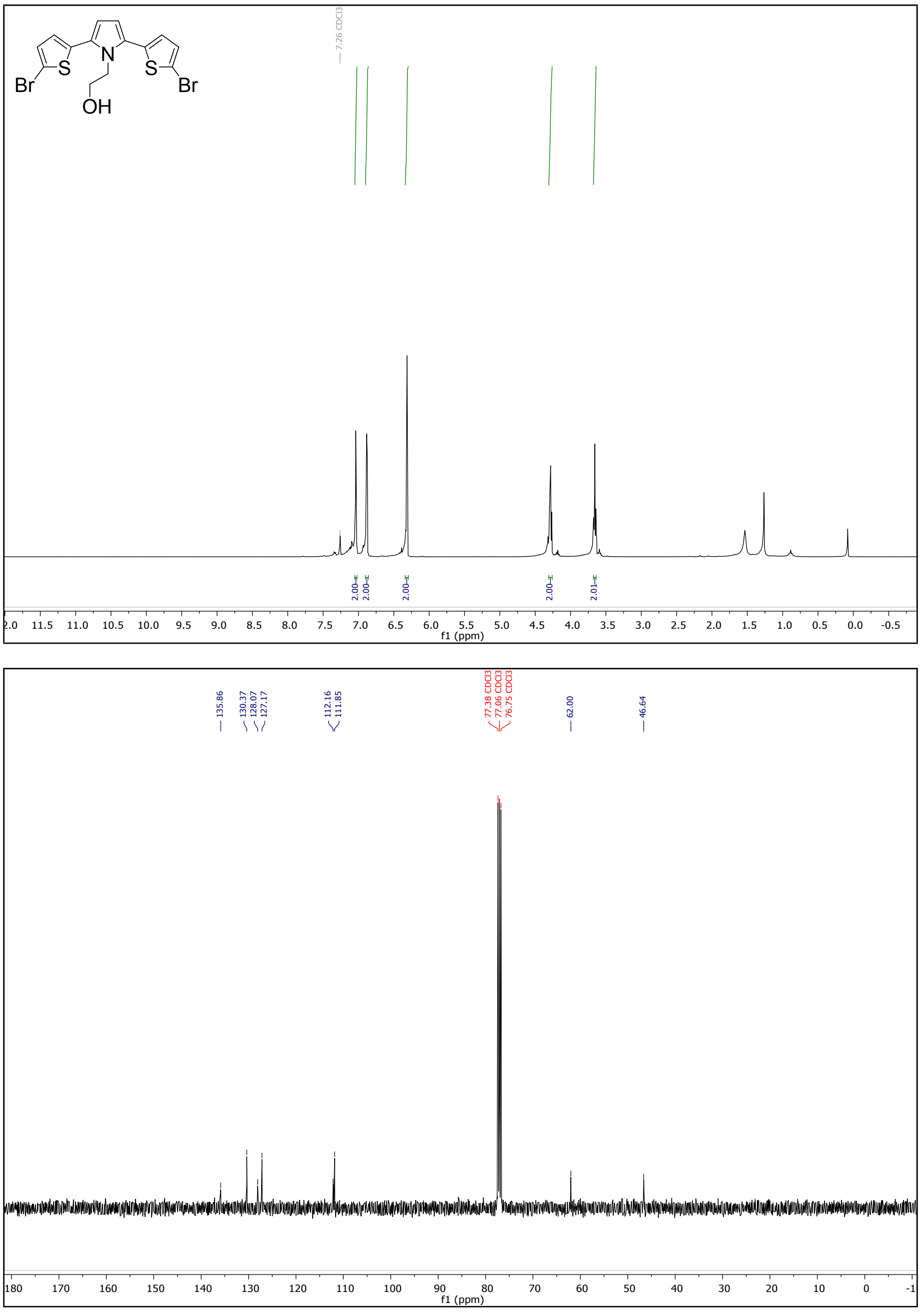

Figure S6. ^{1}H and ^{13}C NMR Spectra of 2-(2,5-bis(5-Bromothiophen-2-yl)-1H-pyrrol-1-yl)ethan-1-ol (2)

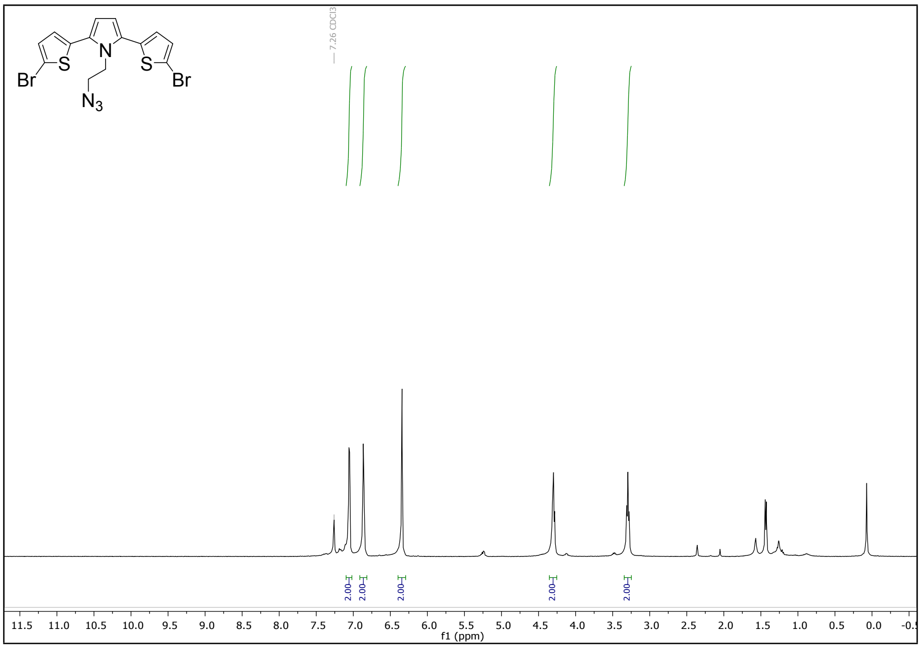

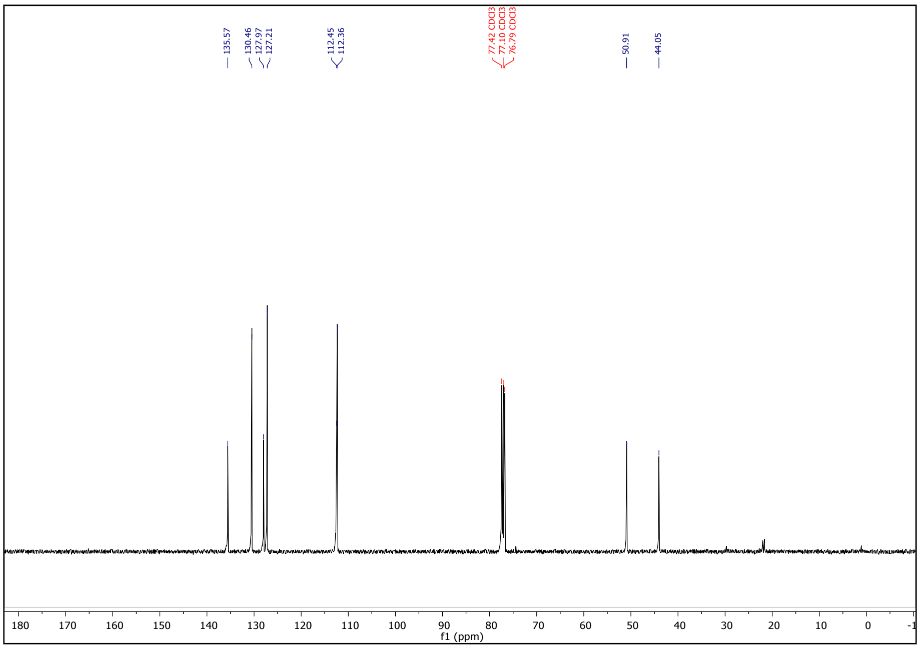

Figure S7. ^{1}H and ^{13}C NMR Spectra of 1-(2-Azidoethyl)-2,5-bis(5-bromothiophen-2-yl)-1H-pyrrole (3)

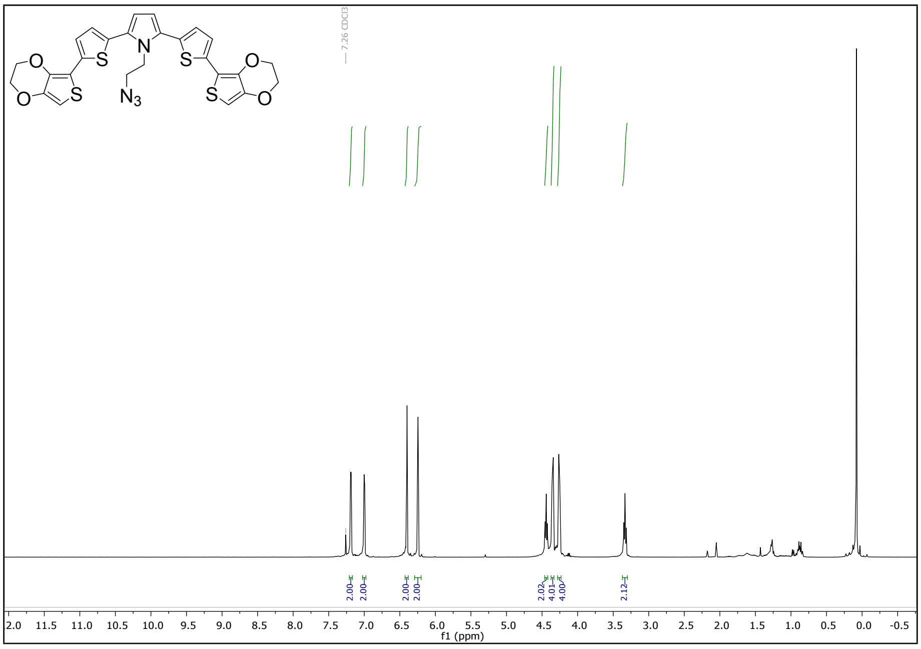

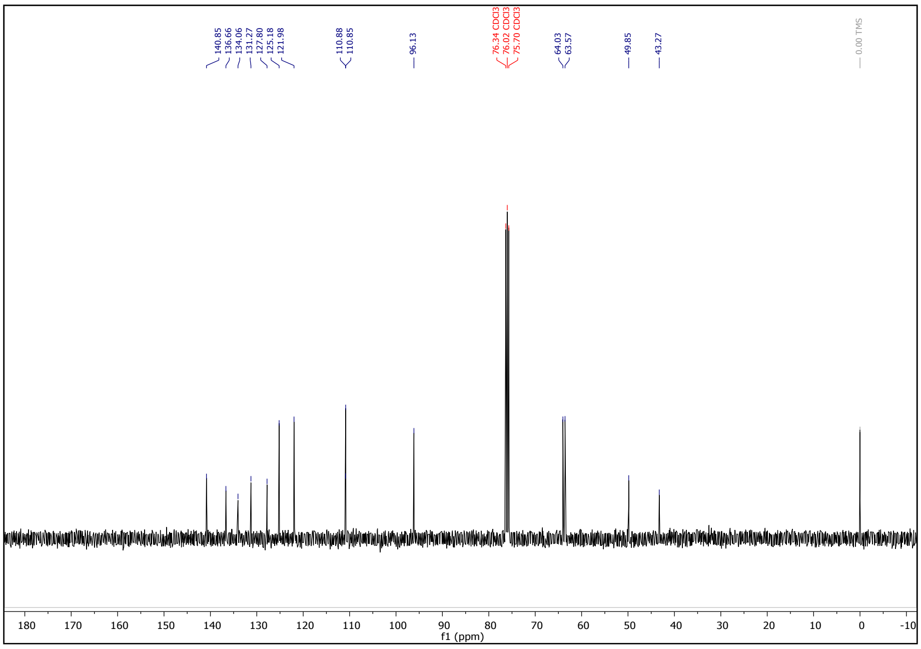

Figure S8. ^1H and ^{13}C NMR Spectra of 1-(2-Azidoethyl)-2,5-bis(5-(2,3-dihydrothieno[3,4-*b*][1,4]dioxin-5-yl)thiophen-2-yl)-1*H*-pyrrole (4)

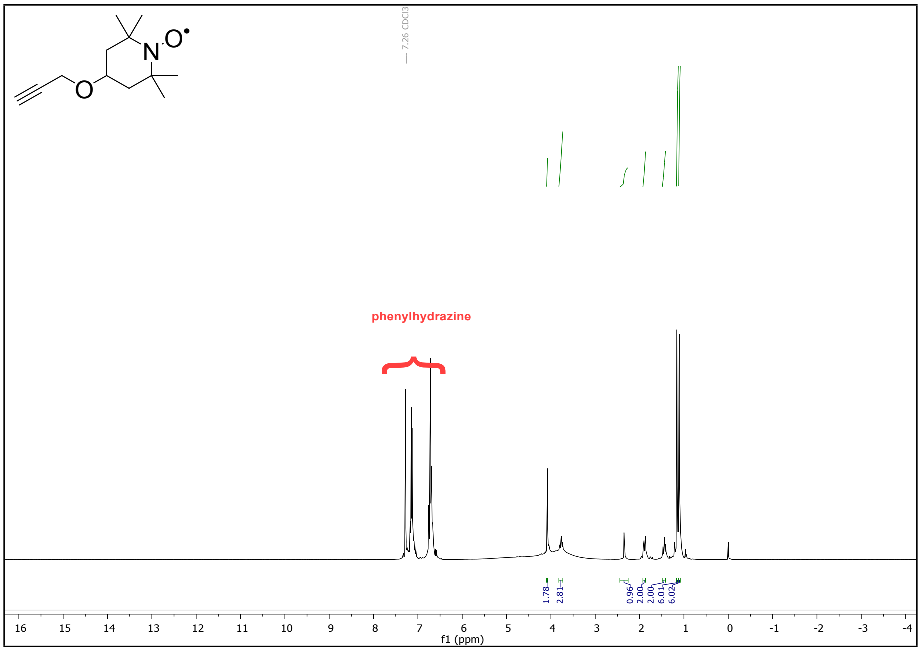

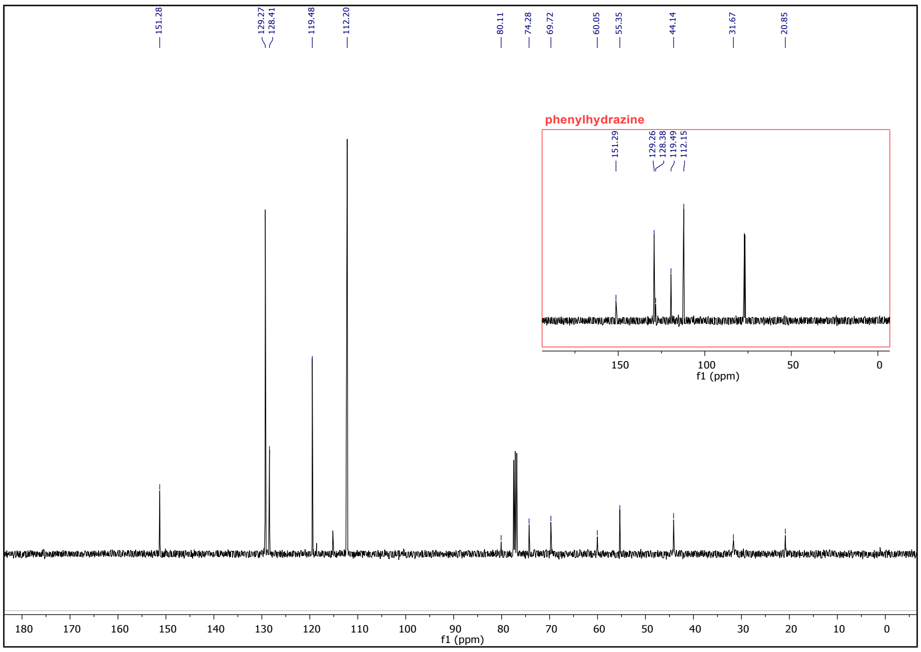

Figure S9. ^{1}H and ^{13}C NMR Spectra of 4-Propargyloxy-TEMPO 5

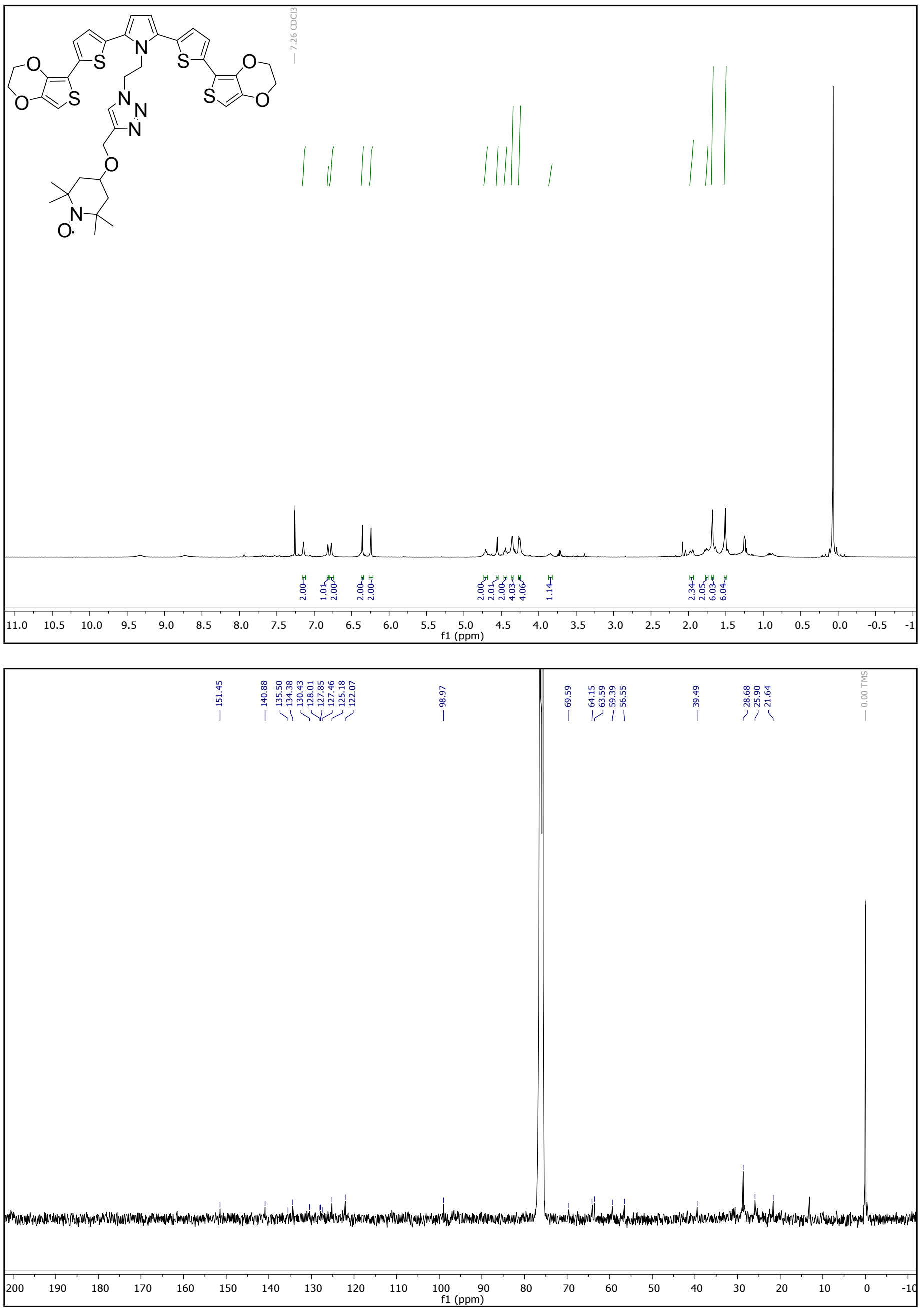

Figure S10. ^{1}H and ^{13}C NMR Spectra of E-SN(T)S-E monomer 6
